# Spatial gradients in cell wall composition and transcriptional profiles along elongating maize internodes

**DOI:** 10.1186/1471-2229-14-27

**Published:** 2014-01-14

**Authors:** Qisen Zhang, Roshan Cheetamun, Kanwarpal S Dhugga, J Antoni Rafalski, Scott V Tingey, Neil J Shirley, Jillian Taylor, Kevin Hayes, Mary Beatty, Antony Bacic, Rachel A Burton, Geoffrey B Fincher

**Affiliations:** 1 Australian Research Council Centre of Excellence in Plant Cell Walls, School of Agriculture, Food and Wine, University of Adelaide, 5064 Adelaide, South Australia, Australia; 2 Australian Research Council Centre of Excellence in Plant Cell Walls, School of Botany, University of Melbourne, 3010 Parkville, Victoria, Australia; 3 Genetic Discovery Group, Crop Genetics Research and Development, Pioneer Hi-Bred International Inc. 7300 NW 62nd Avenue, 50131-1004 Johnston, IA, USA; 4 Genetic Discovery Group, DuPont Crop Genetics Research, DuPont Experimental Station, Building E353, 198803 Wilmington, DE, USA

**Keywords:** Cell walls, Cellulose, Cereals, Lignin, Polysaccharides, Transcription factors

## Abstract

**Background:**

The elongating maize internode represents a useful system for following development of cell walls in vegetative cells in the Poaceae family. Elongating internodes can be divided into four developmental zones, namely the basal intercalary meristem, above which are found the elongation, transition and maturation zones. Cells in the basal meristem and elongation zones contain mainly primary walls, while secondary cell wall deposition accelerates in the transition zone and predominates in the maturation zone.

**Results:**

The major wall components cellulose, lignin and glucuronoarabinoxylan (GAX) increased without any abrupt changes across the elongation, transition and maturation zones, although GAX appeared to increase more between the elongation and transition zones. Microarray analyses show that transcript abundance of key glycosyl transferase genes known to be involved in wall synthesis or re-modelling did not match the increases in cellulose, GAX and lignin. Rather, transcript levels of many of these genes were low in the meristematic and elongation zones, quickly increased to maximal levels in the transition zone and lower sections of the maturation zone, and generally decreased in the upper maturation zone sections. Genes with transcript profiles showing this pattern included secondary cell wall *CesA* genes, GT43 genes, some β-expansins, UDP-Xylose synthase and UDP-Glucose pyrophosphorylase, some xyloglucan endotransglycosylases/hydrolases, genes involved in monolignol biosynthesis, and NAM and MYB transcription factor genes.

**Conclusions:**

The data indicated that the enzymic products of genes involved in cell wall synthesis and modification remain active right along the maturation zone of elongating maize internodes, despite the fact that corresponding transcript levels peak earlier, near or in the transition zone.

## Background

Maize stover, which consists of the residual stalks and leaves of maize plants (*Zea mays*) that remain in the field after harvest, is becoming an increasingly important source of lignocellulosic biomass for second generation bioethanol production. Compositional analyses indicate that dried maize stover, which is composed almost completely of cell wall residues, contains high levels of cellulose, lignin and glucuronoarabinoxylans (GAX) [1]. However, there is relatively little information available either on the distribution of these wall polymers in the mature plant or on their biosynthesis, re-modelling and degradation during normal growth and development. Similarly, little is known about the regulation of wall polysaccharide synthesis in vegetative tissues of maize.

Here, we have measured changes in cell wall composition along an elongating maize internode, which represents a useful model system for the examination of walls of the stalk at different stages of development. Thus, the basal region of the internode includes the intercalary meristem, above which is an elongation zone where cell expansion occurs and primary cell walls are deposited [2,3]. Further up the internode is the transition zone, where cell elongation slows and secondary wall synthesis begins. At the upper or distal end of the internode is the maturation zone, where cells have stopped growing and secondary wall deposition predominates [2]. The compositions of isolated walls in this developmental series have been monitored in 1 cm sections from the bottom of the internode. In parallel, microarray analyses have been used to define gene transcript profiles along the elongating internode and have enabled changes in wall composition to be reconciled with the transcriptional activities of genes involved in wall polysaccharide, protein and lignin synthesis, together with transcription factor genes that are known to regulate the expression of genes responsible for wall synthesis and re-modelling.

Cellulose accounts for about 50% of total dry matter in the mature stalk of maize and is synthesized by members of a family of cellulose synthase (CesA) enzymes, for which there are at least 12 genes in the maize genome [4]. Cellulose is a linear polysaccharide of (1,4)-linked β-d-glucopyranosyl residues with a degree of polymerization from 2000 and up to 14,000, depending on the source [5,6]. Cellulosic chains adopt extended ribbon-like conformations that allow the individual molecules to align into fibrillar aggregates that are stabilized by extensive intermolecular hydrogen bonding and van der Waals interactions, and exhibit high tensile strength. It has been variously suggested that each microfibril consists of 24 or 36 individual cellulose molecules [7,8].

The other major cell wall polysaccharide of maize stover is glucuronoarabinoxylan (GAX) [1]. The GAX components of walls in the grasses generally consist of a backbone of (1,4)-linked β-d-xylopyranosyl residues, some of which are substituted with single α-l-arabinofuranosyl residues at C(O)3 and to a lesser extent at C(O)2. The backbone β-d-xylopyranosyl residues can also be substituted at the C(O)3 position with α-d-glucuronopyranosyl residues or their 4-O-methyl ethers, or with short oligosaccharide chains. Hydroxycinnamic acid residues, in particular feruloyl residues, can be attached to the α-l-arabinofuranosyl residues [6,9,10].

Cell wall composition and properties are dynamic, insofar as they change during normal growth and development, and in response to abiotic and biotic stresses [11]. In young, expanding cells the wall is relatively thin and is highly hydrated. The so-called primary cell is deposited during this developmental phase. The orientation of newly synthesized cellulosic microfibrils is transverse to the axis of the cell elongation [12], which constricts lateral expansion of the cell but allows turgor-driven cell expansion along the elongation axis. When cell expansion slows, the so-called secondary wall is formed, but it is likely that this transition occurs over a period of time rather than as an abrupt event. During the transition and maturation stages of cell development, cellulose, non-cellulosic polysaccharide and lignin concentrations of the wall increase and the structures of non-cellulosic polysaccharides can be altered to meet the changing functional requirements of the wall. These processes are of fundamental importance in allowing anisotropic growth in plant cells and, more specifically, for the elongation of maize internodes.

It has been estimated that approximately 1,200 genes encoding polysaccharide synthases, glycosyltransferases, glycosyl hydrolases and a range of ancillary and regulatory proteins are involved in plant cell wall biosynthesis, remodelling and degradation [13,14]. Some information is also emerging on regulatory genes that participate in wall biology [15]. In Arabidopsis, secondary wall-associated NAC (for NAM, ATAF1/2 and CUC2) domain protein 1 (SND1) regulate suites of target transcription factors, including SND2, SND3, MYB20, MYB42, MYB43, MYB52, MYB54, MYB69, MYB85, MYB103 and KNYT7 (a knotted1-like homeodomain protein) [16,17]. More recently, interacting MYB75 and KNAT7 transcription factors have been shown to modulate secondary cell wall deposition both in stems and seed coat in Arabidopsis [18]. In poplar (*Populus trichocarpa*), the wood-associated transcription factors (PtrWNDs) are the functional orthologs of Arabidopsis SND1 [19]. They regulate a cascade of downstream transcription factors and control the biosynthesis of secondary walls. Pine MYB1 and MYB4 transcription factors and eucalyptus MYB2 regulate lignin biosynthesis by binding to the AC elements of promoters of lignin biosynthetic genes [20-23].

Regulation of wall synthesis and remodelling is also exerted at the protein level, where phosphorylation is an important mechanism for the regulation of enzymic activities. Phosphorylation of the AtCesA1 and AtCesA7 cellulose synthases has been reported in Arabidopsis [24,25], where phosphorylation of AtCesA1, an enzyme that has been implicated in cellulose synthesis in primary walls, affects polar interaction with microtubules [25], while phosphorylation of AtCesA7, a secondary wall CesA, promotes the degradation of this enzyme.

In this study, the changes in cell wall polysaccharide compositions along an elongating maize internode have been compared with mRNA levels for genes encoding enzymes involved in cell wall metabolism, and a number of strong correlations have been observed in the various zones of the internode. For example, transcription of a several transcriptional factor and protein kinase genes is tightly correlated with the transcription of maize *CesA10*, *CesA11* and *CesA13* genes and with lignin metabolic genes.

## Results

### The tenth internode of a maize stalk was divided into ten sections for analysis

When the 10th internode of a maize stalk was 10 cm in length, it was harvested and cut into 10 sections of approximately 1 cm each. At this time the internode was actively elongating and would normally elongate to about 15 cm within a couple of days [26]. The basal Section S1 contained intercalary meristem cells. Sections S2 and S3 were the most actively elongating and are thus designated as the elongation zone here. Cell elongation persisted in Sections S4 and S5, but the elongation rate decreased, particularly in Section S5. We have designated Sections S4 and S5 as the transition zone. Cell elongation had almost or completely ceased in Sections S6 to S10 and substantial lignin deposition was observed. Sections S6–S10 were designated the maturation zone. Overall, the development and morphological appearance of the 10th internode of the maize stalk were as described previously by Morrison *et al.*[26], Kende *et al.*[2] and Bosch et al. [27].

### Crystalline cellulose and lignin increase along the elongating internode

**Figure 1:**
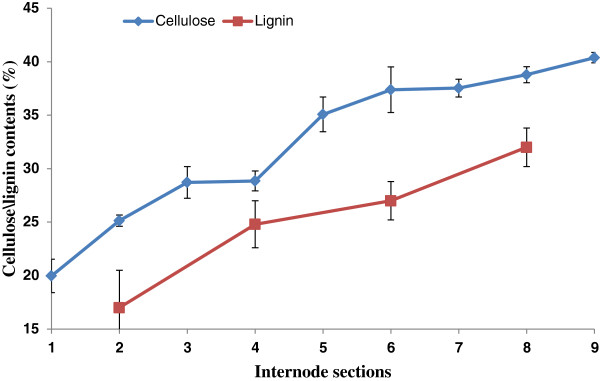
**Concentrations of crystalline cellulose and lignin in sections of the elongating maize internode.** Cellulose (% by weight) was assayed using the acetic acid/nitric acid method of Updegraff [28] from three biological replicates. Lignin was assayed after the AIR was hydrolysed with 25% acetyl bromide in acetic acid and the residual lignin materials were weighed on filter paper and absorbance measured at 280 nm (Hatfield *et al.*) [29]. The data are means of three biological replicates and standard errors are indicated. Lignin was not measured in Sections S1, S3, S5, S7 or S9.

Crystalline cellulose, as determined by acetic-nitric acid analyses [28] was relatively low in Section S1 of the elongating internode, with a value of about 20% (w/w) of the de-starched alcohol-insoluble residue (AIR) of the cell walls (Figure 1). Thereafter crystalline cellulose concentration increased to 25% w/w in Section S2 and continued to increase to about 40% w/w in the distal sections of the internode (Figure 1). Lignin concentrations followed a similar pattern along the elongating internode, showing a steady increase from about 16% w/w in the meristematic zone to about 32% w/w in the maturation zone (Figure 1). Thus, although the lignin and crystalline cellulose approximately doubled on a weight basis from the base to the top of the internode, no dramatic increases in crystalline cellulose or lignin were observed in particular regions of the internode (Figure 1). However, there was an apparent flattening of the curve between Sections S3 and S4, and the rate of increase of crystalline cellulose appeared to taper off after Section S6 (Figure 1).

### Substantial changes in wall polysaccharide composition and fine structure occur along the internode

**Figure 2:**
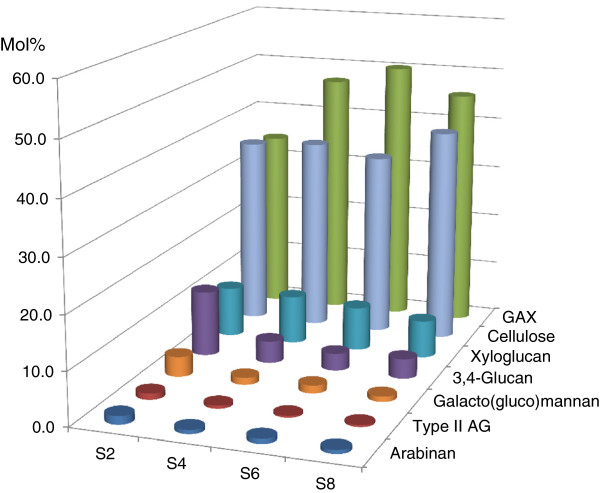
**Polysaccharide compositions in the elongating maize internode.** Polysaccharides were deduced from neutral sugars after methylation analysis. S2: Section S2; S4: Section S4; S6: Section S6 and S8: Section S8. The data are averages of three biological replicates.

Compositional and linkage analyses of the de-starched alcohol-insoluble residues (AIR) of the maize internode sections (Additional file 1: Table S1) allowed the proportions of the major wall polysaccharides to be deduced [30-32]. Cellulose estimated in these analyses ranged from about 36% mol/mol in the basal sections to 41% mol/mol in the maturation zone (Figure 2). Thus, comparison of the cellulose contents estimated by the method of Updegraff [28], which measures crystalline cellulose (Figure 1), and the methylation analyses, which estimate total cellulose (Figure 2), suggested that cellulose crystallinity increased from the elongation zone, through the transition zone, to the maturation zone.

The trend in the GAX contents of the walls was generally similar to that of cellulose, although GAX appeared to increase more between the elongation and transition zones. In the elongation zone, about 34% GAX was detected and this rose to about approx. 46% in the Section S8 of the maturation zone (Figure 2). Together, these two polysaccharides accounted for between 70 and 86% of the walls in the elongating maize internode. Other wall polysaccharides were also detected but were each generally less than 10% of the total. In each case these were more abundant in the elongation zone (Section S2 of Figure 2) and decreased to relatively low levels in the maturation zone (Section S8 of Figure 2). For example, the (1,3;1,4)-β-glucan concentration decreased from about approx. 12% to 4% from the base to the top of the internode (Figure 2).

Other minor components of the wall preparation, including pectic arabinan, arabinogalactan-proteins and galactomannans, also decreased from the base to the top of the internode (Figure 2). Overall, these developmental patterns of wall polysaccharides along the elongating maize internode were remarkably similar to those observed for developing barley coleoptiles, except that higher levels of pectic polysaccharides were detected in the walls of young barley coleoptiles [30].

### Global gene transcription patterns vary significantly along the elongating internode

**Figure 3:**
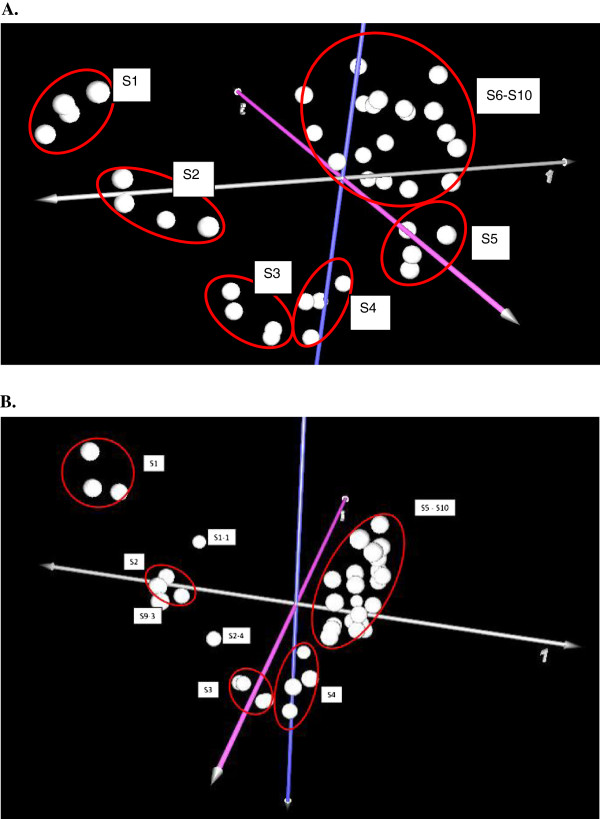
**Principal component analyses of internode microarray data.** All genes **(A)** and genes involved in cell wall synthesis, re-modelling and degradation **(B)**. MultiExperiment Viewer software was used for PCA and construction of the graphs (http://www.tm4.org). S1-1: Section S1 replicate 1; S2-4: Section S2 replicate 4; S9-3: Section S9 replicate 9.

Principal component analysis (PCA) of microarray data for all genes revealed three properties of gene transcription profiles of the samples (Figure 3A). Firstly, sample replicates were clustered in single groups, indicating that sample preparation was reproducible and the microarray data were reliable for further analysis. Secondly, transcription patterns differed in the sections containing the meristem, the elongation zone and the transition zone. Thirdly, transcripts from Sections S6 to S10 of the maturation zone formed a single cluster (Figure 3A).

Of the 17,200 functionally annotated genes on the maize microarray, about 5% (930) encoded genes have been implicated in cell wall polysaccharide synthesis, lignin synthesis, or in metabolic pathways leading to the synthesis of wall constituents. When these genes were subjected to PCA analysis, they grouped into clusters that were similar to those observed for the entire gene set, although the S5 cluster merged with the S6-S10 cluster (Figure 3B). As expected, the data revealed spatial differences in the transcription patterns of genes associated with wall synthesis, re-modelling and degradation in meristematic, elongation and transition zones, but less pronounced differences between the transition and maturation zones (Figure 3B).

The very different transcription patterns of genes involved in wall biology along the internode were further demonstrated through comparisons that showed transcript abundance of about 40% of cell wall genes transcribed in any section changed by two-fold or more compared with Section S1 (column 2 of Table 1). The exception to this pattern was seen between Sections S1 and S2, where transcript levels of relatively fewer ‘cell wall’ genes changed (Table 1). Sequential comparisons between adjacent internode sections showed that differential transcription of cell wall genes decreased from the lower to the upper regions of the internode (Table 1).

**Table 1:** Differential expression of cell wall genes in an elongating maize internode

**Sections**	**Number of genes changed by 2-fold or more (%)**	**% of differentially transcribed genes between any two sections**							
		**S2**	**S3**	**S4**	**S5**	**S6**	**S7**	**S8**	**S9**
S2	214 (23)								
S3	418 (45)	54							
S4	390 (42)	56	15						
S5	370 (40)	52	27	26					
S6	389 (42)	54	32	30	13				
S7	408 (44)	61	30	27	30	27			
S8	389 (42)	56	34	33	18	10	24		
S9	372 (40)	58	33	31	16	10	22	10	
S10	386 (41)	59	34	34	26	20	22	15	23

The 4 × 44 microarray chips contained 17200 putative functional genes. About 5% (930 genes) of them was found to code for cell wall metabolic proteins. While Section S1 was taken as the control, the number of genes with a change in signal intensity by 2- or more than 2-fold (both down and up-regulated) was calculated for other sections (showing in column 2). The percentage overall cell wall genes was listed in brackets. The differential transcription between any two adjacent sections was also calculated and is shown in columns 3 – 10.

### Transcription profiles of CesA genes show different patterns along the internode

**Figure 4:**
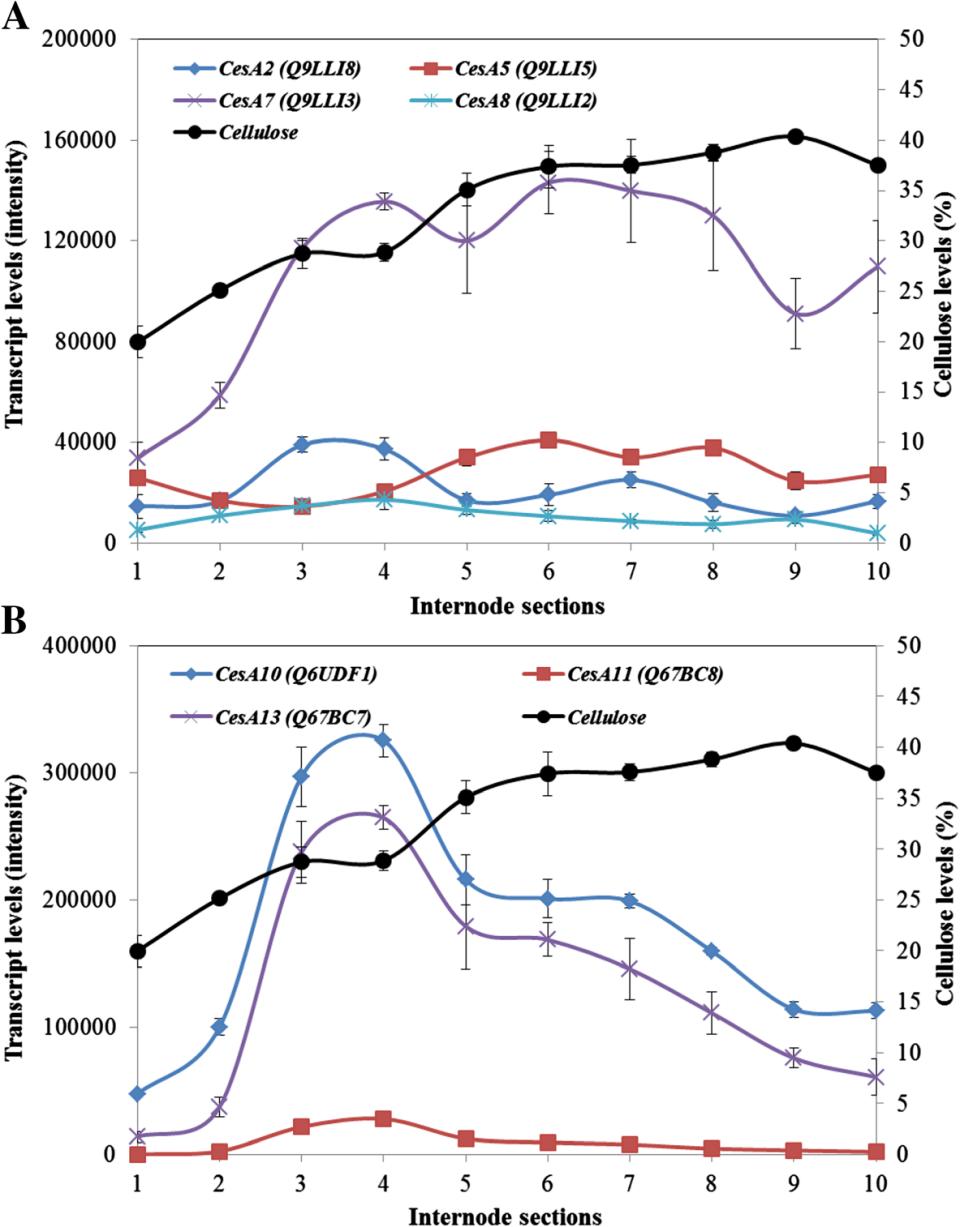
**Transcript levels of *ZmCesA *genes involved in primary (A) and secondary (B) cell wall synthesis in the elongating maize internode.** The data are averages of four biological replicates with indications of standard errors. Uniprot accession numbers for the genes are indicated in brackets. Cellulose levels in the individual sections are also indicated.

The maize genome contains at least 12 *CesA* genes, some of which are believed to be involved in cellulose synthesis during primary cell wall deposition (group 1), while others are involved in cellulose synthesis during secondary wall deposition (group 2) [4]. The transcript levels of representative *ZmCesA* genes from the primary wall group 1 were generally quite low compared with those of the secondary cell wall group 2 genes (Figure 4A cf. Figure 4B) and did not change much from the bottom to the top of the internode (Figure 4A). However, transcripts of the *ZmCesA7* gene were detected at levels similar to those for the other primary wall *CesA* genes in the meristematic and lower elongation zone sections, but increased to relatively high levels in the transition zone and remained high throughout the maturation zone (Figure 4A).

The *CesA* genes from group 2 showed quite different transcription patterns. In each case transcript abundance increased from low levels in Section S1 to high levels in the transition zone Sections S3 and S4, before decreasing steadily in the maturation zone Sections S6-S10 (Figure 4B). These data are consistent with the apparent flattening out of crystalline levels after Sections S5 and S6 (Figure 1). Overall, levels of *ZmCesA11* (Figure 4B) and *ZmCesA12* (not shown) transcripts were low. Transcript profiles of these *ZmCesA* genes were checked by quantitative real-time PCR (QPCR) and showed good correspondence with the microarray data (Additional file 1: Figure S1).

### Transcription profiles of cellulose synthase-like genes also show variable patterns

**Figure 5:**
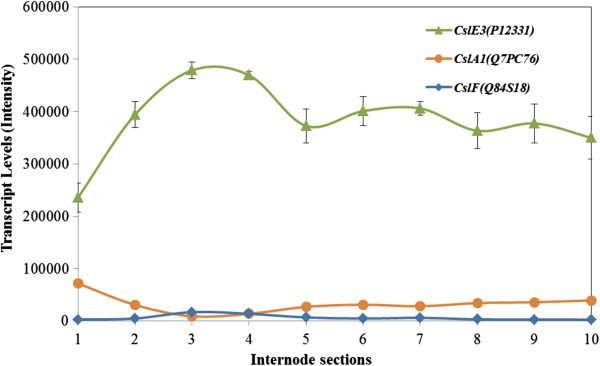
**Transcript levels of *CSLA*, *CSLF *and *CSLE *genes in the elongating maze internode.** Statistical treatment was as for Figure 4.

Cellulose synthase-like (*Csl*) genes were underrepresented on the microarray, but transcript levels of the few *ZmCsl* genes that were annotated and highly transcribed are compared in Figure 5. Of these, the *ZmCslE3* mRNA was most abundant in the maize internode and approached or exceeded levels recorded for the most abundant *CesA* transcripts (Figure 5). In contrast to most of the *CesA* transcripts, levels of the *ZmCslE3* transcripts did not decrease significantly between Sections S5 and S10 (Figure 5). The functions of the *CslE* group of genes have not yet been defined. The transcript pattern of the *ZmCslA1* gene was quite characteristic, insofar as transcripts were high in the meristematic Section S1, decreased rapidly in the elongation zone, and subsequently increased again through the transition and maturation zones in Sections S3 to S10 (Figure 5). At least some members of the *CslA* gene sub-family are believed to encode mannan synthases [33,34]. Another *ZmCslA* gene was present on the microarray but its transcript levels were 40- to 200-fold lower than those of *ZmCslA1* (Additional file 1: Figure S2). The transcript pattern of the *ZmCslA3* gene was very similar to the characteristic pattern observed for the *ZmCslA1* gene (Additional file 1: Figure S2).

Two *ZmCslF* genes were represented on the microarray. Their transcript levels were generally low but relatively higher in Sections S3 and S4 (Figure 5 and Additional file 1: Figure S2). The maize genome is believed to have at least an additional five *CslF* and several *CslH* genes, but these were not represented on the microarray. Two *ZmCslD* genes were represented on the microarray, but their transcript levels were extremely low (data not shown).

### Glycosyl transferase genes implicated in wall synthesis were differentially transcribed along the internodes

**Figure 6:**
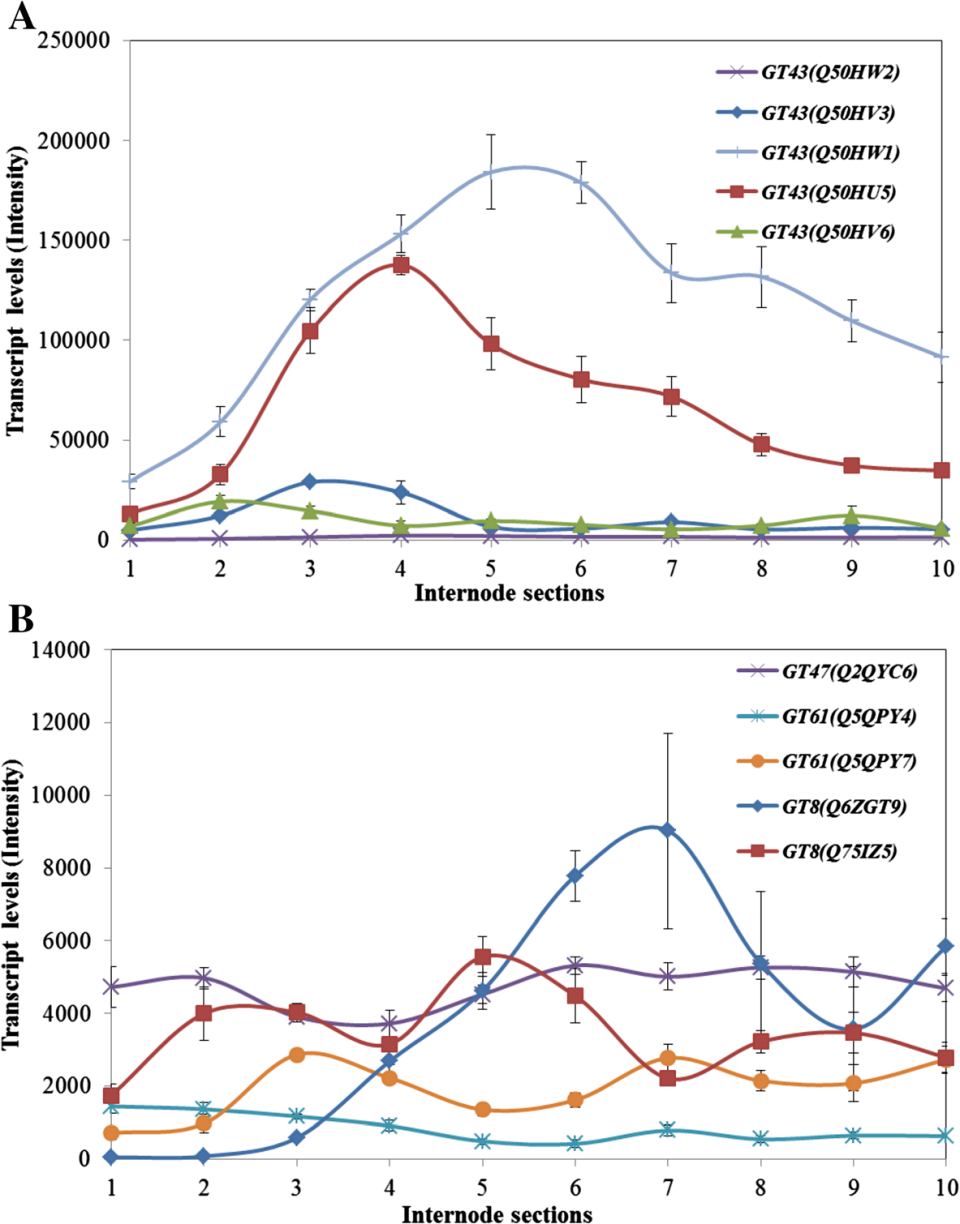
**Transcript levels of genes implicated in GAX synthesis, including families GT43 (A), GT8, GT47 and GT61 (B) in the elongating maize internode.** Statistical treatment was as for Figure 4.

In addition to the GT2 genes described in the sections above, several other glycosyl transferase (GT) gene families have been implicated in cell wall polysaccharide biosynthesis. In particular, members of the GT8, GT43, GT47 and GT61 (http://www.cazy.org/) [35] families of genes are believed to be involved in GAX synthesis in higher plants [36-41]. Accordingly, members of these gene families that were represented on the microarray were monitored in the ten sections along the 10th maize internode. Of these, transcript levels were highest for the two GT43 genes (Figure 6A) and transcript abundance generally peaked in Sections S4 to S6, which include the elongation and transition zones, but in some cases transcripts were high in the lower sections (Figure 6A). Levels of transcripts for the GT8, GT47 and GT61 genes were relatively low in all sections of the internode (Figure 6B).

### Genes involved in wall expansion and re-modelling are abundantly transcribed in all zones of the internode

**Figure 7:**
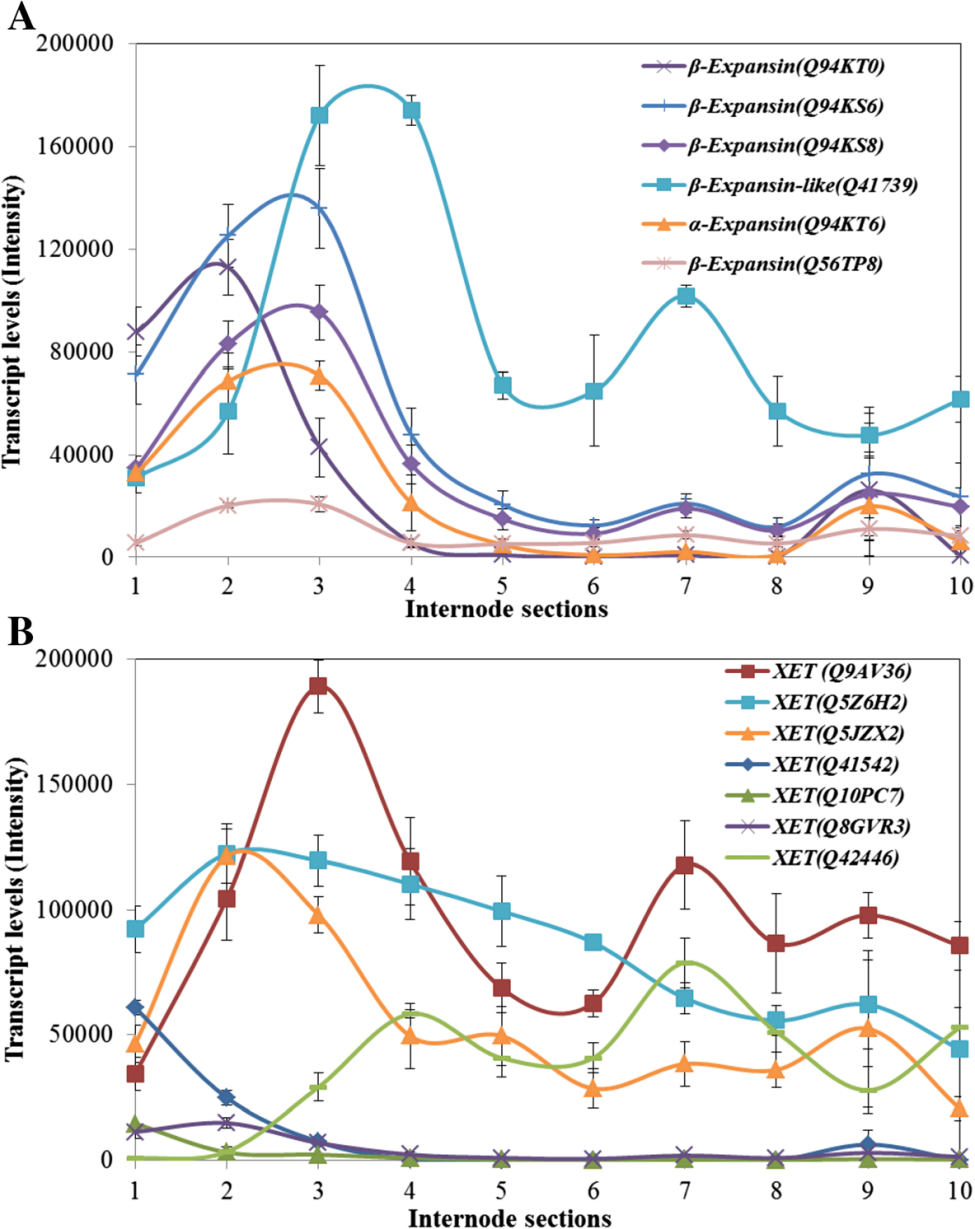
Transcript levels of expansin (A) and XET (B) genes in the elongating maize internode.

Transcription of members of the expansin gene families was monitored in sections of the 10th maize internode using the microarrays. The expansins are involved in auxin-induced cell elongation [42,43] and also play important roles in the anisotropic gravitropic responses in maize [44]. Several β-expansin and one α-expansin genes were represented on the microarray and their transcript levels are shown in Figure 7A. Transcripts were very high in the elongation zone, consistent with their function as wall loosening agents during cell elongation. In these cases, transcript levels peaked in Sections S3 and S4 (Figure 7A).

Xyloglucan endotransglycosylases/hydrolases (XET) are believed to play a role in auxin-induced cell elongation [42,45]. Their functions include modification of existing polysaccharide structures, in particular xyloglucans, and possibly the covalent cross-linking of different polysaccharides such as xyloglucans, cellulose and (1,3;1,4)-β-d-glucans [46-48]. Several putative XET genes were found on the microarray. The relative abundance of mRNA from the different XET genes varied widely, as did their transcription patterns (Figure 7B). In some cases, transcript levels were relatively high in the meristematic tissue of Section S1 and in other cases specific XET gene transcript levels remained high from the basal to the distal ends of the internode (Figure 7B).

### Key sugar nucleotide interconversion gene transcripts peak in the transition zone

**Figure 8:**
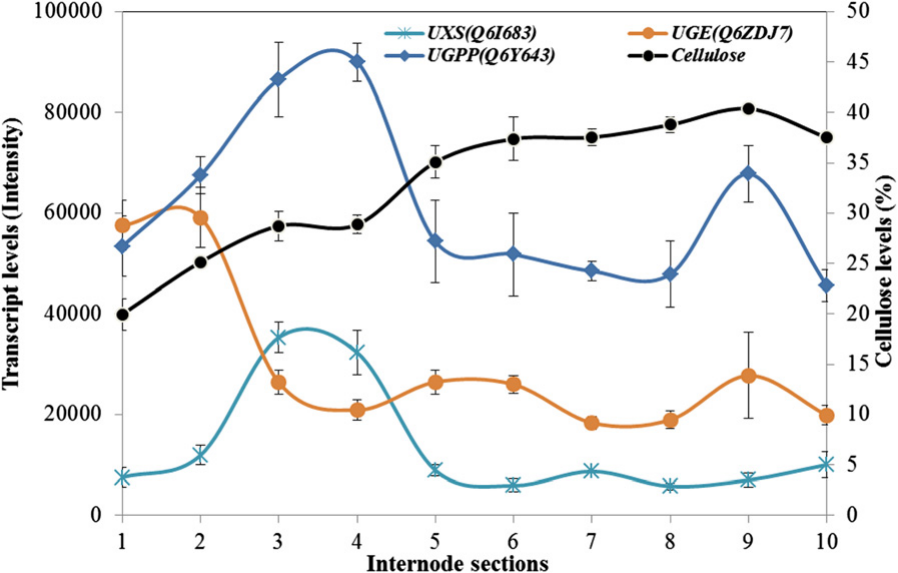
**Transcript levels of genes involved in sugar nucleotide interconversions in the elongating maize internode.** These include UDP-xylose synthase (UXS), UDP-glucose epimerase (UGE) and UDP-glucose pyrophosphorylase (UGPP) genes. Cellulose levels are also indicated.

Biosynthesis of the major cell wall polysaccharides in maize stalks requires sugar nucleotides such as UDP-Glc, UDP-GlcA, UDP-Xyl and UDP-Ara, and the transcript levels of important genes in the interconversion pathways were therefore examined in the maize internode sections. The UDP-Glc pyrophosphorylase (UGPP) enzyme controls the entry of carbon into the sugar nucleotide pools through the formation of UDP-Glc from glucose 1-phosphate and UTP. Transcript levels of one of the maize *UGPP* genes (Q6Y643) was very high in the meristematic tissues of Section S1, and increased substantially in Sections S2–S4 of the elongation and transition zones (Figure 8). Another important gene in the sugar nucleotide interconversion pathways is UDP-Xyl synthase (UXS), which catalyses the irreversible decarboxylation of UDP-GlcA to form UDP-Xyl. The UXS enzyme therefore commits carbon to the formation of pentose sugars, which are major constituents of the maize GAX polysaccharide. Transcript levels of one of *UXS* genes (Q6J683) were about 5-fold lower than those for the *UGPP* gene, but showed a similar pattern along the internode. The UXS gene transcripts also peaked in Sections S3 and S4 of the transition zone (Figure 8).

### High transcription levels of polysaccharide endohydrolase genes were detected

**Figure 9:**
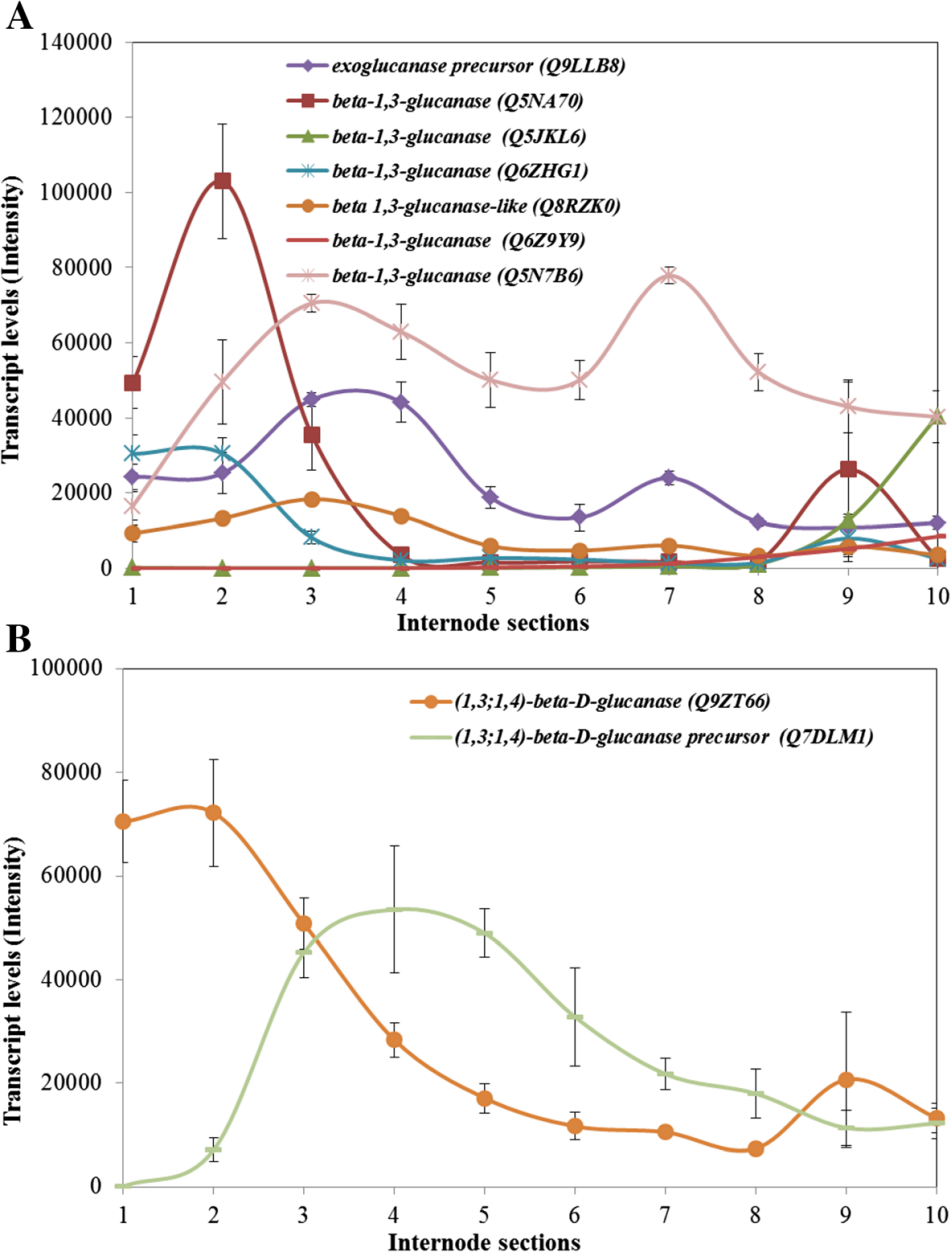
**Transcript levels of selected polysaccharide hydrolase genes in the elongating maize internode.** These include β-glucan exohydrolase and(1,3)-β-glucanase **(A)** and (1,3;1,4)-β-glucanase genes **(B)**.

There is a good deal of evidence that cellulose synthesis requires the participation of the hydrolytic enzyme (1,4)-β-glucanase, or cellulase, and Zhou *et al.*[49] showed that this class of enzyme affects internode elongation in rice. Similarly, (1,3;1,4)-β-glucan endohydrolases have been implicated not only in (1,3;1,4)-β-glucan depolymerisation, but also in (1,3;1,4)-β-glucan synthesis [50]. Levels of transcripts for the corresponding polysaccharide endohydrolase genes, together with (1,3)-β-glucanase genes, were therefore monitored along the maize internode (Figure 9). Transcripts of a cellulase gene were not particularly high in any region of the internode, but certain (1,3)-β-glucanase genes were actively transcribed in the elongation and transition zones, with transcripts for one of these genes also detected along the maturation zone (Figure 9A). Transcripts for two (1,3;1,4)-β-glucanase genes had quite different patterns along the internode. In one case (gene Q9ZT66), transcripts were highest in the meristematic tissues of Section S1, but decreased steadily through the elongation, transition and maturation phases (Figure 9B). This pattern is consistent with the reduction of (1,3;1,4)-β-glucan levels in walls of the internode from the lower sections to the top of the internode (Figure 2). However, transcripts of a second (1,3;1,4)-β-glucanase gene (Q7DLM1) were not detected in Section S1, but increased in abundance to a peak in the transition zone Sections S3 and S4 (Figure 9B).

### Lignin pathway gene transcripts were high in the transition and lower maturation zones

**Figure 10:**
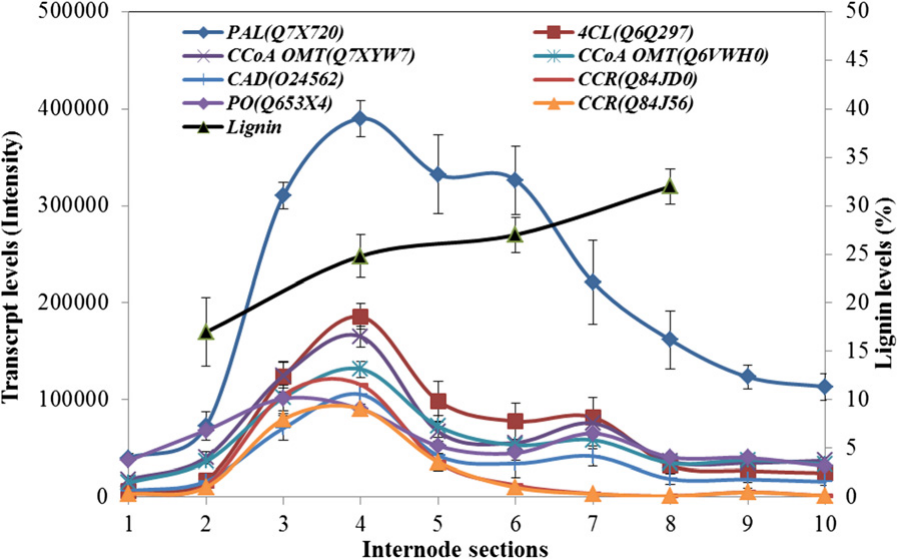
Transcript levels of lignin biosynthetic genes in the elongating maize internode.

More than 120 probes on the microarray represented genes involved in lignin metabolism. Transcript levels of many of these lignin metabolism genes were low in the meristematic Section S1 and in the early transition zone sections, but quickly rose to relatively high levels with peaks in the transition zone Sections S3 and S4. Thereafter, transcript levels generally decreased to relatively low levels in the top Section S10 of the maturation zone (Figure 10). The genes included phenylalanine ammonia-lyase (PAL, Q7X720), 4-coumarate-CoA ligase (4CL, Q6Q297), two caffeoylCoA 3-O-methyltransferase genes (CCoA-OMT, Q7XYW7 and Q6VWH0), cinnamyl alcohol dehydrogenase (CAD, Q24562), peroxidase (PO, Q653X4) and two cinnamoyl-CoA reductase genes (CCR, Q84JD0 and Q84J56) (Figure 10). The mRNA of the PAL gene (Q7X720) was the most abundant along the internode and also was of maximal abundance in the transition zone (Figure 10).

A few other lignin-related genes showed increased transcription along the internode sections and remained high in the maturation zone (Additional file 1: Figure S3). These included two CCR genes (Q84JG9, Q24563), three peroxidase genes (Q43416, Q5I3F1, Q9ZTF7) and ferulate 5-hydroxylase (F5H, Q109F2). One of the lignin metabolism genes (Q8W2X2) had relatively high transcript levels in Sections S1 and S2 (Additional file 1: Figure S3), but low transcript levels in the transition and maturation zones.

### Transcription factor gene transcripts were detected

**Figure 11:**
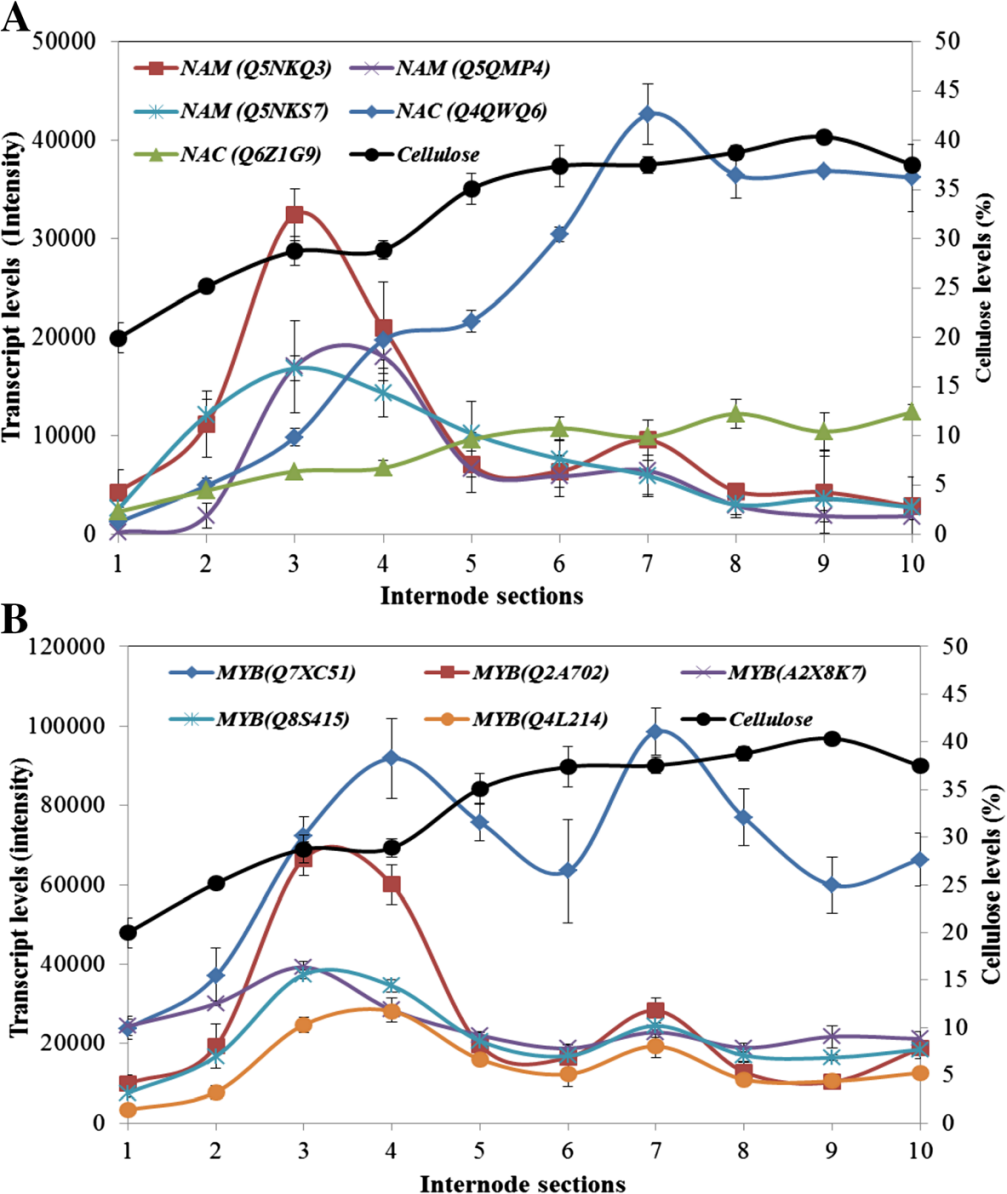
**Transcript levels of transcription factor genes in the elongating maize internode.** NAM family **(A)** MYB family **(B)**. Cellulose levels are also indicated.

The NAC-domain genes encode plant-specific transcription factors and regulate a wide range of activities including organ differentiation, development, plant disease and stress tolerance. Some NAC-domain transcription factors play a very important role in the initiation of secondary cell wall biosynthesis in Arabidopsis and poplar [16,17,19,51]. The maize internode microarray contained more than 30 NAC-domain probes, most of which showed very low transcript levels in the elongating internode. However, three NAM genes (Q5NKQ3, Q5QMP4 and Q5NKS7) showed an elevated transcription in the elongation and transition zones (Figure 11A). Another two NAC-domain genes (Q4QWQ6 and Q6Z1G9) had different transcriptional patterns, with low transcript levels in the intercalary meristem and increasing levels in the elongation zone that reached a plateau level in the maturation zone (Figure 11A).

The MYB transcription factors have diverse functions in the regulation of gene transcription and a few of this class are believed to be targets of the NAC-domain transcription factors [52-54]. More than 100 putative MYB transcription factors annotations were found on the microarray. Eleven of these had very high transcript levels in the elongation internode (Figure 11B). The mRNA levels of 4 MYB genes (Q2A702, A2X8K7, Q8S415 and Q4L214) were low in Section S1, increased in Section S2, peaked at either Section S3 or Section S4, decreased in Section S5 and remained low in Sections S6 to S10 (Figure 11). Transcription activity of the MYB gene (Q7XC51) was also low in Section S1, increased in Section S2, peaked at Section S4, decreased in Section S5 and S6, and peaked again in Section S7. The transcript level of this MYB gene was most abundant among all MYB genes (Figure 11B).

## Discussion

When cell wall compositions were determined in ten sections of the elongating tenth internode of V16 stage maize plants, it was apparent that crystalline cellulose levels increased from about 20% by weight in the lower meristematic regions of the internode to over 40% in the upper S9 section of the maturation zone, although the rate of increase decreased after Section S6 (Figure 1). The cellulose contents estimated from methylation-based linkage analyses as (1,4)-glucosyl residues [31,32] were generally higher than the values for crystalline cellulose (compare Figure 1 and Figure 2) and would include both crystalline and non-crystalline cellulose (cf. [13]), but the proportions of crystalline cellulose were decreased in the lower, younger regions of the internode. Thus, approximately 70% crystalline cellulose was measured in Section S2 and 95% in Section S8. Lignin also showed a similar steady rate of increase in the internodes, from about 15% by weight in the lower sections to about 30% by weight in the upper regions of the maturation zone. In neither case were any abrupt changes observed in cellulose or lignin concentrations as the developmental stage of the internode cells passed from meristematic tissues, through the elongation zone, transition zone and the maturation zone. In absolute terms, Scobbie *et al.*[3] showed that total cell wall concentrations were higher in the maturation zone than in lower sections of the internode.

The major non-cellulosic polysaccharide in wall preparations from the ten internode sections was GAX, which was estimated from the linkages analyses based on previously published structures of these polysaccharides [31]. Levels of GAX in the isolated walls were about 32% in the elongation zone Section S1, but increased sharply to about 45-50% in the transition and maturation zones (Figure 2). An important characteristic of the cell wall is that the plant can adjust the physicochemical properties of constituent polysaccharides and hence their function in the wall. For example, in the case of GAX, the degree of substitution of the (1,4)-β-xylan backbone can be altered. A lower degree of substitution will enable chains to form intermolecular hydrogen bonding with the (1,4)-β-xylan backbone of other GAX molecules, or with cellulose [10]. Here, the degree of substitution of the xylan backbone with arabinosyl residues decreased markedly along the internode, but in particular between the elongation zone and the transition zones. The average Xyl:Ara ratios in the GAX, taken from three biological replicates, was 1.6:1 for Section S2, 6.6:1 for Section S4, 8.2:1 for Section S6 and 7.5:1 for Section S8 (data not shown). From these values, one would predict that the solubility of the GAX would decrease dramatically along the internode and that the ability of the GAX to interact with other polysaccharides through molecular alignment and intermolecular hydrogen bonding, would greatly increase, especially between the elongation and transition zones. From the overall colorimetric analyses of uronic acids in the sections, it was possible to estimate that the degree of substitution of the xylan backbone of the GAX with GlcA residues was about 30% in Section S2 of the elongation zone, but this decreased to about 10% in the transition zone and maturation zone Sections S4, S6 and S8 (data not shown). The overall uronic acid levels of 3.5% to 5% by weight of the internode were comparable with the values of 4% reported for barley husk [10]. These uronic acid contents are deduced to be primarily GlcA and hence present on the GAXs as terminal residues, because carboxyl reduction/methylation analyses of the elongation zone (data not shown) showed negligible levels of either terminal- or 4-GalA residues, which suggested that only low levels of pectic galacturonans/RG1 were present, consistent with the virtual absence of Rha residues in the sugar analyses.

Minor polysaccharides of the walls included xyloglucans, (1,3;1,4)-β-glucans, galactomannans, arabinans and type II arabinogalactans. These showed similar changes along the elongating internode; in each case their levels decreased steadily from the lower to the upper regions of the internode, again without any abrupt changes between the different developmental stages represented by the elongation, transition and maturation zone sections (Figure 2).

Attempts were subsequently made to reconcile the changes in compositions of cell walls described above with changes in gene expression, as measured by transcript abundance in the ten sections of the elongating internode. In undertaking these comparisons, it is important to note that transcript abundance will not necessarily be directly related to levels of the encoded enzymes and, similarly, will not always be proportional to the levels of polysaccharide products of the synthase enzymes. Nevertheless, many consistent trends were observed between transcript levels and cell wall composition. Microarray techniques were applied to this question and when both total gene transcripts and the levels of transcripts of genes that can be linked with cell wall metabolism were analysed, easily discerned differences in transcript profiles between the internode sections became apparent. Principal component analyses (PCA) highlighted these differences (Figure 3A cf. Figure 3B) and we conclude from these data that it is necessary to divide the elongating maize internode into sections to obtain a more complete understanding of changes in gene transcription at different developmental stages. This approach may be contrasted with that of Bosch *et al.*[27] who compared elongating and non-elongating whole maize internodes for their microarray experiments.

Cellulose biosynthesis is catalysed by at least two groups of CesA enzymes. One group is believed to be responsible for cellulose synthesis in the primary wall and the other for secondary wall cellulose synthesis [55]. In the maize genome, 12 *CesA* genes have been identified [4]. Transcripts of *CesA10*, *CesA11* and *CesA12* genes are abundant in vascular bundles and these have been implicated in secondary wall cellulose synthesis [4]. The maize genomic DNA sequence has revealed another secondary wall *CesA* gene, which had a similar sequence with *CesA12* gene and is designated *CesA13*.

Transcript levels for the *ZmCesA* gene family were determined along the elongating tenth internode and particular attention was paid to transcript levels of *ZmCesA* genes that have previously been implicated in cellulose synthesis either in primary or secondary walls [4]. One might expect that transcripts for primary wall *CesA* genes would have been highest in the meristematic and elongation regions of the internode and to have subsequently decreased in the transition and maturation zones, but this was not observed. Of the *ZmCesA* genes representative of those proposed to be involved in primary wall cellulose biosynthesis, most showed relative low levels of transcript right along the internode (Figure 4A). The notable exception here was the *ZmCesA7* gene, for which relatively high transcript levels were detected in the transition and maturation zones of the internode (Figure 4A). At this stage we are unable to explain the transcript pattern for the *ZmCesA7* gene.

Transcript levels for the secondary cell wall *ZmCesA* genes were relatively low in the meristematic tissues and in the elongation zone, as expected, but increased quickly in the upper elongation zone and the transition zone to show a quite distinct peak in the transition zone, whereafter they steadily decreased (Figure 4B). Similarly, transcript levels for genes encoding enzymes involved in monolignol biosynthesis and other steps in lignin biosynthesis [56] were low in the lower sections of the internode, as expected, but increased to a marked peak in the transition zone before steadily decreasing in the maturation zone (Figure 10). These characteristic transcription patterns for the cellulose synthase and lignin synthesis genes must be reconciled with the steady increases of both cellulose and lignin from the lower elongation zone to the upper maturation zone of the internode (Figure 1). One explanation would be that the CesA enzymes and those involved in lignin synthesis are relatively stable and, once synthesised, can continue to generate cellulose and lignin over several days in the maturation zone. Another possibility is that a pool of lignin monomers is generated and that their polymerization into lignin continues over several days. In the case of cellulose, the formation of crystalline forms might lag behind the synthesis of the nascent cellulose chains, although the decrease in secondary wall *CesA* mRNA levels in the maturation zone (Figure 10) matches the slower rate of increase in cellulose after Sections S5 and S6 (Figure 1). Whatever the explanation, it is clear that the peaks and troughs of transcript levels observed here in the different regions of the internode are ‘flattened out’ to allow a steady increase in crystalline cellulose and lignin from the bottom to the top of the elongating internode (Figure 1).

The developmental patterns of transcripts of genes that are believed to mediate GAX synthesis were somewhat harder to interpret, mainly because our current understanding of heteroxylan synthesis is incomplete. Nevertheless, a number of genes have been shown to be involved. Members of the GT43 and GT47 families of glycosyl transferases are thought to be involved in the elongation of the xylan backbone [36,38,41,57,58], GT8 enzymes have been shown to be involved in glucuronosyl substitution of the xylan chain [39,59,60] and GT61 enzymes are likely to be arabinosyl or xylosyl transferases [61,62]. The most abundant transcripts from these groups of genes were those encoding GT43 enzymes, which showed developmental patterns (Figure 6A) that were remarkably similar to those of the secondary cell wall cellulose synthases (Figure 4B). In both cases transcripts peaked in the transition zone and in the lower maturation zone. These observation are consistent with suggestions that the GT43 enzymes mediate (1,4)-β-xylan chain elongation. However, levels of gene transcripts for the enzymes believed to be involved in xylan backbone substitution, namely the GT8 and GT61 enzymes, remained low along the entire length of the internode (Figure 6B).

Transcript levels for the UXS gene, which encodes an enzyme that is central to the synthesis of the important GAX sugar donor substrates, namely UDP-Xyl and UDP-Ara, followed a similar pattern to the GT43 gene and the secondary wall *CesA* genes (Figure 8), while the UGPP gene that commits carbon to sugar-nucleotide synthesis more generally, was transcribed at high levels right along the internode, but again with a peak in the transition zone (Figure 8).

Of the genes that have been implicated in the biosynthesis of the minor non-cellulosic wall polysaccharides, *CslE3* gene transcripts were the most abundant; the functions of CslE enzymes have not been defined. Surprisingly high levels of *CslA1* transcripts were detected in the meristematic tissues of Section S1, but these decreased quickly in the elongation zone before rising again during maturation (Figure 5). As noted earlier, *CslA* genes are believed to be involved in mannan and glucomannan synthesis [33,34], but there is only about 3% (w/w) mannan in the walls in the elongation zone, and this decreases to and remains at very low levels thereafter (Figure 2 and our unpublished data). It has been observed elsewhere that mannan levels and *CslA* transcripts are relatively high at the early stages of development of barley endosperm [63] and barley coleoptiles [30], but the data suggest that mannans might be more important in the early stages of wall deposition in very young tissues.

During the elongation phase of anisotropic cell growth, a number of enzymes and proteins, including expansins and xyloglucan endotransglycosylases (XETs), are believed to mediate in the wall loosening process that is considered essential for cell expansion to occur [42]. Transcripts of genes encoding both classes of proteins were detected at high levels along the maize internode (Figure 7). Many of the expansin gene transcripts preceded the secondary wall *CesA* and lignin-associated genes (Figure 7A cf. Figure 4A and Figure 10), with peak levels in the elongation zone. This is consistent with the expansion of cells in those zones of the internode. In contrast, the patterns of XET transcript development were highly variable and could not be easily linked with changes in internode developmental stages or wall composition (Figure 7B). The XET gene families in plants are generally large [64] and the enzymes have been implicated not only in xyloglucans modification, but also in the modification or covalent cross-linking of cellulose and various non-cellulosic wall polysaccharides [46-48,65]. Participation in the re-modelling of a range of wall polysaccharides would be consistent with the highly variable transcript profiles observed in the elongating maize internode (Figure 7B).

Other genes that have been implicated in wall re-modelling, but also in turnover and degradation, include polysaccharide hydrolases. For example, high levels of transcripts for a β-glucan exohydrolase were observed, with maximal levels in the transition zone (Figure 9A). The β-glucan exohydrolase is a non-specific exo-acting enzyme that is capable of hydrolysing a wide range of oligoglucosides and might be required to completely degrade various β-glucans to their constituent sugars [49]. In contrast, one of the (1,3;1,4)-β-glucanase genes decreased steadily from the lower internode regions to the upper maturation zone (Figure 9B). This paralleled the decreases in (1,3;1,4)-β-glucan concentration in the wall along the internode (Figure 2). However, another (1,3;1,4)-β-glucanase gene was transcribed in the transition zone and lower maturation zone (Figure 9B), when the levels of (1,3;1,4)-β-glucan were very low indeed. Transcripts of several genes encoding (1,3)-β-glucanases were also detected (Figure 9A), but these enzymes are encoded by a very large gene family and perform a wide range of functions in plants [66], consistent with the range of developmental patterns seen in the elongating maize internode (Figure 9A).

It is noteworthy that the same five β-expansin genes and one of the XET genes (Q5JZX2) observed here also play a role in gravitation-induced cell elongation in the maize pulvini [44]. The transcript levels of these genes increase substantially in the lower pulvini, where cell elongation is induced by gravitational stress [44]. The (1,3;1,4)-β-glucanase and the β-1,3-glucananase genes are also showed to be involved in gravitation-induced cell elongation in the lower region of maize pulvini [44]. Thus, it appears that the genes identified in the present study through their high transcript levels in the normal growth and development of elongating maize internodes are also transcribed in response to gravitational stress.

Given the similarities in transcription patterns for many of the genes discussed above, a global correlation analysis was performed and gene transcripts that were highly correlated with the secondary cell wall *ZmCesA* genes are listed in Table 2. Most of these genes had Pearson co-efficiency values close to or greater than 0.9 and included genes involved in non-cellulosic wall polysaccharide synthesis, remodelling and depolymerisation, together with genes that mediate lignin synthesis. Correlations were also seen with several transcription factor genes and protein kinase genes (Table 2). Of particular interest amongst the transcription factor genes were the NAM (Q5QMP4) gene and the MYB (Q2A702) gene, because these showed developmental patterns that were similar to those seen for many of the *CesA*, lignin and glycosyl transferase genes (Figure 10A and Figure 10B). Several MYB and NAM transcription factors from Arabidopsis have been shown to control secondary wall biosynthesis [17,52] and coordinated transcription of NAM and secondary wall *ZmCesA* genes was also observed in gravitationally stressed maize pulvini [44].

**Table 2:** Correlation analysis of cell wall genes in the elongating maize internode

**Genes**	**CesA10**	**CesA11**	**CesA13**	**At gene**	**Expect**	**At name**
**Cell wall metabolism**						
CesA10 (Q6UDF1)	1.00	0.91	0.89	At5g44030	0	AtCesA4
CesA11 (Q67BC8)	0.91	1.00	0.91	At4g18780	0	AtCesA8
CesA13 (Q67BC7)	0.97	0.89	1.00	At5g17420	0	AtCesA7
BK2 (A1DZD8)	0.90	0.96	0.88	At5g15630	0	AtIRX6
BK2 (A0EJ90)	0.95	0.97	0.90	At5g15630	0	AtIRX6
Glycosyltransferase (Q50HU5, GT43 family)	0.96	0.94	0.95	At5g67230	E-173	AtIRX14H
Glycosyltransferase (Q50HW1, GT43 family)	0.71	0.92	0.92	At2g37090	E-93	AtIRX9
3-β-glucuronosyltransferase-like (Q5QM25)	0.85	0.94	0.88	At1g27600	E-91	AtIRX9H
(1,3;1,4)-β-glucanase (Q7DLM1, GT17)	0.79	0.93	0.85	At4g16260	E-102	-
**Lignin metabolism**						
PAL (Q7X720)	0.91	0.84	0.95	At2g37040	0	AtPAL1
Laccase (Q10ND7)	0.89	0.95	0.92	At5g60020	0	AtLAC17
Laccase (Q2PAJ0)	0.74	0.95	0.93	At5g60020	0	AtLAC17
O-methyltransferase (Q6VWG5)	0.90	0.94	0.89	At5g54160	E-116	AtOMT1
Caffeoyl CoA 3-O-methyltransferase (Q7XYW7)	0.90	0.94	0.85	At4g34050	E-118	AtCCoAOMT1
Caffeoyl CoA 3-O-methyltransferase (Q6VWH0)	0.91	0.96	0.88	At4g34050	E-104	AtCCoAOMT1
Cinnamyl alcohol dehydrogenase (O24562)	0.90	0.94	0.87	At3g19450	E-177	AtCAD4
4-coumarate coenzyme A ligase (Q6Q297)	0.93	0.92	0.87	At3g21240	0	At4CL2
Peroxidase P7X (Q9ZTS7)	0.72	0.91	0.76	At5g05340	E-82	-
Carboxylate oxidase (Q75IP9)	0.74	0.90	0.90	At1g17020	E-79	AtSRG1
**Transcription factor and protein kinases**						
Nucellin-like aspartic protease (Q5Z6M6)	0.95	0.97	0.90	At1g49050	E-114	-
Putative blue copper protein (Q6Z7U7)	0.91	0.98	0.85	At1g49050	E-114	-
Cdc protein kinase-like (Q653F8)	0.83	0.96	0.88	At5g67210	E-62	-
LEM3 (Ligand-effect modulator 3)-like (Q653D8)	0.94	0.93	0.90	At1g79450	E-106	AtLEM3
NAM (Q5QMP4)	0.93	0.95	0.93	At4g28500	E-99	AtSND2
Knox (Q94LW3)	0.94	0.95	0.87	At1g62990	E-109	AtIRX11
BAG (Q5N9K2)	0.93	0.92	0.94	At3g51780	E-40	AtBAG4
POT family protein (Q2R726)	0.84	0.93	0.90	At1g27040	E-83	-
VP1/ABI3 (Q6Z3U3)	0.78	0.92	0.85	At4g32010	E-152	AtHSL1
MYB (Q0J3I9)	0.81	0.90	0.83	At5g67300	E-54	AtMYBR1
F-box (Q5VR67)	0.84	0.91	0.79	At2g26850	E-79	-
Protein kinase (Q2RBK1)	0.96	0.92	0.94	At1g56720	E-150	-
Putative NEP1-interacting protein (Q6Z2U9)	0.85	0.92	0.90	At1g74410	E-42	-
Serine/threonine-protein kinase SAPK3 (Q75V63)	0.88	0.91	0.92	At4g33950	E-142	AtOST1
PREG-like (Q6Z2N6)	0.78	0.90	0.79	At2g44740	E-62	AtCycP4;1

Pearson correlation co-efficient (P-) values were calculated for the genes showing high correlations in transcription activities with secondary wall *CesA10* (Q6UDF1), *CesA11* (Q67BC8)**,** and *CesA13* (Q67BC7) genes. Arabidopsis genes (At) that are orthologous to the maize genes are identified in the right hand columns.

High correlations were also observed between the secondary wall *ZmCesA* gene transcripts and various protein kinases (Table 2). Although the roles of these kinases in the regulation of cellulose synthesis have not been examined further here, it seems likely from other studies that phosphorylation or de-phosphorylation of CesA enzymes affects their activities. For example, phosphorylation of the Arabidopsis AtCesA7 protein, a subunit of the secondary wall CesA complex, targets this protein for a degradation, while mutation of the phosphorylation sites of the Arabidopsis AtCesA1 enzyme, which is involved in primary wall cellulose synthesis, affects polar interactions between the CesA complex and microtubules [24,25]. In the elongating maize internode, transcription of two protein kinases (Q653F8 and Q75V63) is highly correlated with transcription of secondary wall *CesA* genes (Table 2).

## Conclusions

Analyses of wall compositions and transcript profiles in 1 cm sections of the elongating 10th internode of maize plants revealed several unexpected developmental patterns. Firstly, the three major cell wall components, cellulose, GAX and lignin, increased relatively steadily without any abrupt changes between the elongation, transition and maturation zones of the elongating internode. Secondly however, transcript profiles of genes known to be involved in the synthesis or re-modelling of these major wall components did not match the steady increases in cellulose, GAX and lignin mentioned above. Instead, transcript levels of many of these genes were low in the meristematic and elongation zones, quickly increased to maximal levels in the transition zone and lower sections of the maturation zone, and generally decreased in the upper maturation zone sections towards the top of the internode. Genes with transcript profiles showing this pattern included secondary cell wall *CesA* genes, GT43 genes, some β-expansins, UXS and UGPP, cellulase, most of the genes in metabolic pathways leading to the synthesis of monolignols, and NAM and MYB transcription factor genes. The fact that transcription of many of these genes decreased along the maturation zone, where the levels of cellulose, GAX and lignin continued to increase, suggested that the enzymic products of the genes remained active right along the maturation zone, despite the fact that the corresponding transcript levels had declined. Furthermore, the analyses enabled us to identify transcription factors and protein kinases that are potentially involved in the regulation of cell wall synthesis and the functions of some of these genes are currently under investigation.

## Methods

### Plant material

Maize plants (O9B) were grown in a glasshouse at 17/27°C under natural light conditions. An elongating internode (about 10 cm in length) from the V16 developmental stage of maize [67] plants was harvested, cut into 10 sections and frozen immediately in liquid nitrogen for cell wall polysaccharide and mRNA preparation.

### Cell wall preparation and analysis

Cell wall polysaccharides were determined as described previously [44]. Maize stalk sections were ground under liquid nitrogen, exhaustively extracted with 80% v/v ethanol and the residue (AIR) thoroughly de-starched with pancreatic α-amylase [31]. The ethanol-insoluble residues (de-starched AIR) were dried by solvent exchange with methanol and acetone, and stored in a vacuum desiccator with dried silica gel. Crystalline cellulose was determined by the acetic acid/nitric acid method of Updegraff [28], with modifications as described in Pettolino *et al.*[31]. The procedure was accurate to approximately 3%. Lignin was determined colorimetrically by the acetyl bromide method as described by Hatfield *et al.*[29] with the following modifications. Approximately 2 mg cell wall material (in duplicate) was hydrolysed with 0.5 mL 25% acetyl bromide in acetic acid (v/v) for 2.75 h at 70ºC, followed by the addition of 4.4 mL of a premixed solution of 10 mL 2 M NaOH with 12 mL acetic acid, made up to 10 mL by weight. Absorbance was measured at 280 nm and lignin calculated based on an extinction coefficient of 20 ^gL-1cm-1^.

Monosaccharide linkage analysis was performed in duplicate by methylation with methyl iodide in sodium hydroxide and DMSO as described by Ciucanu and Kerek [68] followed by hydrolysis, reduction and acetylation and data calculated as mol% of total AIR, as previously described [31,32]. Monosaccharide linkages (mol%) and relative polysaccharide proportions were deduced from the partially methylated alditol acetates that were separated and analysed by GC-MS as previously described [31,32]. Using these procedures and the known structures of wall constituents, it is possible to assign individual partially methylated alditol acetate derivatives, or portions thereof, to different wall polysaccharides. For example, 4-linked glucosyl residues can be assigned to xyloglucan, cellulose and/or (1,3;1,4)-β-glucan, while galactosyl residues might be assigned to pectins, arabinogalactan-proteins, xyloglucans, etc. A complete description of the formulae used for these calculations is provided in Pettolino *et al.*[31].

Uronic acids were assayed according to Filisetti-Cozzi and Carpita [69] with some modifications. Briefly, d-glucuronic acid was used as the calibration standard (1 mg/mL). Sample tubes were prepared by the addition of 0.4 mL water in 10 mL tubes at 4°C, and 80 μL 4 M sulfamic acid-potassium sulfamate (pH 1.6) and 2.4 mL 75 mM sodium tetraborate in concentrated H_2_SO_4_ were added. In a separate tube, approximately 2 mg sample was weighed and hydrolysed in 200 μL 75 mM sodium tetraborate in concentrated H_2_SO_4_. A 20 μL aliquot of the hydrolysed sample was added to the sample tubes containing sulfamic acid-potassium sulfamate and sodium tetraborate. All tubes were pulsed in a centrifuge and heated in a water bath to 90°C for 20 minutes, whilst ensuring the water level did not exceed that within the tubes by more than 1 cm. Tubes were cooled on ice and absorbance was measured at 525 nm. This was followed by the addition of 80 μL 0.15% m-hydroxybiphenyl in 0.5% sodium hydroxide. Tubes were mixed and allowed to rest for 10 min to allow colour to develop, before absorbance was measured at 525 nm.

### Transcript profiling by microarrays

Microarray analyses were conducted as described previously [44]. Maize internode sections were ground under liquid nitrogen and total RNA extracted with the TRIZOL reagent (Invitrogen, Sydney) according to the manufacturer’s instructions. Polyadenylated mRNA was purified using the Illustra mRNA Purification Kit (GE Biosciences). Sample quality and RNA concentration were assessed using an Agilent Bioanalyzer. The mRNA was reverse-transcribed into double-stranded cDNA, which was labelled with Cy3 or Cy5 fluorescent dye, using the Agilent Low RNA Linear Amp kit. Biological replicates were labelled alternately using Cy3 or Cy5 to guard against dye-bias. The cDNA was hybridized to Agilent 4 × 44 k maize gene microarrays [70] and the microarrays were washed according to Agilent standard protocols. The microarray chips were scanned with an Agilent G2505B DNA Microarray Scanner at two laser power settings (100% and 10%). The images were inspected visually for image artefacts, and feature intensities were extracted, filtered and normalized with Agilent Feature Extraction Software (v 9.5.1). The data were normalized using quantile normalization (BOLSTAD http://www.ncbi.nlm.nih.gov/pubmed/12538238). The normalized data were used to compare expression levels of genes related to cell wall compositions.

### Quantitative RT-PCR analysis of maize cell wall genes

Transcription analysis of maize cell wall genes was carried by real time PCR (Q-PCR) according to Burton *et al.*[71]. The same batch of RNA used for the microarray analysis was used for the synthesis of cDNA. The primer sequences used for Q-PCR are listed in Additional file 1: Table S2.

## Competing interests

We cannot identify any financial or non-financial interests associated with the work described in this manuscript.

## Authors’ contributions

QZ: Performed most of the experimental work, together with experimental design and analysis and interpretation of data. RC: Performed the linkage analyses. KSD: Substantial contribution to the conception and design of the work, experimental design and analysis of the data. JAR: Substantial contribution to the analysis and interpretation of the data. SVT: Substantial contribution to the conception of the work, and final approval for publication. NJS: Performed the transcript profiles and analysis of data. JT: Performed a substantial part of the experimental work and analysis of data. KH: Performed the microarray data analyses. MB: Performed the microarray analyses. AB: Analysed the linkage data. RAB: Substantial contribution to the experimental design and analysis of the data. GBF: Substantial contribution to the conception and design of the work, experimental design and analysis of the data. All authors read and approved the final manuscript.

## Supplementary Material

Additional file 1: Table S1 Linkage analyses of cell wall preparations along the 10^th^ elongating maize internode. S2: Section 2; S4: Section 4; S6: Section 6; S8: Section 8. The data are averages of three biological replicates. **Table S2.** PCR primers used for quantitative RT-PCR. **Figure S1.** QPCR analysis of secondary wall *CesA* genes. QPCR was conducted with primers list in Table S2 with the same RNA for the microarray experiment. Similar transcript profiles were obtained for the primary wall *CesA* genes from both QPCR and microarray experiments (Figure 4). **Figure S2.** The mRNA levels of *Csl* genes. **Figure S3.** Transcript levels of genes involved in lignin synthesis. Click here for file
