# Supplementary material for: Spatial gradients in cell wall composition and transcriptional profiles along elongating maize internodes
Source: BMC Plant Biol. 2014 Jan 14;14:27. doi: 10.1186/1471-2229-14-27 (PMC3927872; doi:10.1186/1471-2229-14-27)
Supplement: Additional file 1: Table S1 — Linkage analyses of cell wall preparations along the 10th elongating maize internode. S2: Section 2; S4: Section 4; S6: Section 6; S8: Section 8. The data are averages of three biological replicates. Table S2. PCR primers used for quantitative RT-PCR. Figure S1. QPCR analysis of secondary wall CesA genes. QPCR was conducted with primers list in Table S2 with the same RNA for the microarray experiment. Similar transcript profiles were obtained for the primary wall CesA genes from both QPCR and microarray experiments (Figure 4). Figure S2. The mRNA levels of Csl genes. Figure S3. Transcript levels of genes involved in lignin synthesis. [file 1471-2229-14-27-S1.docx]

**Spatial gradients in cell wall composition and transcriptional profiles along elongating maize internodes**

**Qisen Zhang^1^, Roshan Cheetamun^2^, Kanwarpal S. Dhugga^3^, J. Antoni Rafalski^4^, Scott V. Tingey^4^, Neil J. Shirley^1^, Jillian Taylor^1^, Kevin Hayes^3^, Mary Beatty^3^, Antony Bacic^2^, Rachel A. Burton^1^, Geoffrey B. Fincher^1^***

**Supplemental Tables and Figures**

**Supplemental Table S1** Linkage analyses of cell wall preparations along the 10^th^ elongating maize internode. S2: Section 2; S4: Section 4; S6: Section 6; S8: Section 8. The data are averages of three biological replicates.

|  |  | **S2** |  | **S4** |  | **S6** |  | **S8** |  |
| --- | --- | --- | --- | --- | --- | --- | --- | --- | --- |
|  |  | **Average** | ***SD*** | **Average** | ***SD*** | **Average** | ***SD*** | **Average** | ***SD*** |
| **Xyl(p)** |  |  |  |  |  |  |  |  |  |
|  | terminal | 1.9 | 0.2 | 1.4 | 0.3 | 1.4 | 0.3 | 1.4 | 0.1 |
|  | 2- | 1.6 | 0.2 | 3.4 | 0.2 | 3.2 | 0.5 | 3.0 | 0.1 |
|  | 4- | 11.9 | 0.4 | 30.7 | 2.3 | 32.9 | 3.7 | 29.5 | 3.0 |
|  | 2,4- | 1.4 | 0.2 | 1.6 | 0.8 | 2.2 | 0.9 | 1.6 | 0.6 |
|  | 3,4- | 7.5 | 0.2 | 5.4 | 0.4 | 5.0 | 0.8 | 4.8 | 1.1 |
|  | 2,3,4- | 5.6 | 0.3 | 4.1 | 0.0 | 4.9 | 2.6 | 5.6 | 1.5 |
| **Ara(f)** |  |  |  |  |  |  |  |  |  |
|  | terminal | 7.9 | 0.8 | 5.1 | 0.4 | 5.2 | 1.1 | 4.0 | 0.1 |
|  | 2- | 0.2 | 0.2 | 0.1 | 0.1 | 0.1 | 0.1 | 0.1 | 0.1 |
|  | 3- | 0.2 | 0.2 | 0.1 | 0.1 | 0.1 | 0.1 | 0.1 | 0.1 |
|  | 5- | 0.8 | 0.1 | 0.5 | 0.0 | 0.4 | 0.0 | 0.4 | 0.1 |
|  | 2,5- | 0.1 | 0.1 | 0.1 | 0.1 | 0.1 | 0.1 | 0.1 | 0.1 |
|  | 3,5- | 0.6 | 0.2 | 0.2 | 0.2 | 0.4 | 0.4 | 0.2 | 0.2 |
| **Gal(p)** |  |  |  |  |  |  |  |  |  |
|  | terminal | 0.6 | 0.1 | 0.4 | 0.1 | 0.4 | 0.2 | 0.1 | 0.0 |
|  | 3- | 0.1 | 0.1 | 0.1 | 0.1 | 0.1 | 0.1 | 0.0 | 0.0 |
|  | 6- | 0.1 | 0.1 | 0.1 | 0.1 | 0.1 | 0.1 | 0.1 | 0.1 |
|  | 3,6 | 1.0 | 0.5 | 0.4 | 0.2 | 0.2 | 0.2 | 0.3 | 0.1 |
| **Glc(p)** |  |  |  |  |  |  |  |  |  |
|  | terminal | 0.8 | 0.3 | 0.5 | 0.5 | 0.5 | 0.5 | 0.6 | 0.2 |
|  | 3- | 3.7 | 0.9 | 1.3 | 0.3 | 1.0 | 0.4 | 1.1 | 0.0 |
|  | 4- | 49.2 | 2.6 | 42.1 | 1.6 | 39.5 | 5.5 | 45.0 | 1.4 |
|  | 4,6- | 2.9 | 0.1 | 1.9 | 0.1 | 1.7 | 0.1 | 1.3 | 0.5 |
| **Man(p)** | |  |  |  |  |  |  |  |  |
|  | 4- | 1.9 | 0.5 | 0.7 | 0.0 | 0.7 | 0.1 | 0.5 | 0.0 |

**Supplemental Table S2** PCR primers used for quantitative RT-PCR

| **Gene name** | **Forward** | **Reverse** |
| --- | --- | --- |
| ZmCesA1 | CGAGACAGTGACTCTTATTTGAAG | ACTGCACGCATAACAAAACTTC |
| ZmCesA2 | ATCTGAACAGGCTCAGCCAAAGATC | CCAGCACTACAAGCAAGGTAGAAAG |
| ZmCesA3 | CGAACTGGCTGCTGAATAACATTG | GGTATGCACGACAGTCAACATGAAAC |
| ZmCesA4 | TGGAAGGTTTGTACTTTGTAGAAAC | TGGTAAACAATATCCACTGGTCTGT |
| ZmCesA5 | GATGAGCTGAAGATAGTTAAAGAGTGG | GACCAGCGGAGGTACATATAGCA |
| ZmCesA6 | CCGTCAGTGTGTGGTCGAAGAAG | CAATACTGCACTCACGAGGCTG |
| ZmCesA7 | GCAAGGTCTTTTGATTCTGC | TCCAATGAACAGGCATACAT |
| ZmCesA8 | CTGTGTCCATTGGAGCAGGAGA | TGTCCTTTGTCGCTAATATACAGACTG |
| ZmCesA9 | GGAGGTGGAAGGTTTGTAGA | TAAACGGAGTATCCCCTTGT |
| ZmCesA10 | CTCCATCTTCTCGCTCGTCT | GCCCAAAAGTGCCTGTATGT |
| ZmCesA11 | GTCTCCTCTGTCTATCTCGCATC | GAGCCGAATTTTAACATTTCAGG |
| ZmCesA12 | AATTGCTGAGCTGTTTATTAAGGTTC | GCCATTTAACAATCAAACCTTGTCC |
| ZmCesA13 | GCTGTCTATCACGGTTCAAA | GGGTCCAAGAACATTACACA |


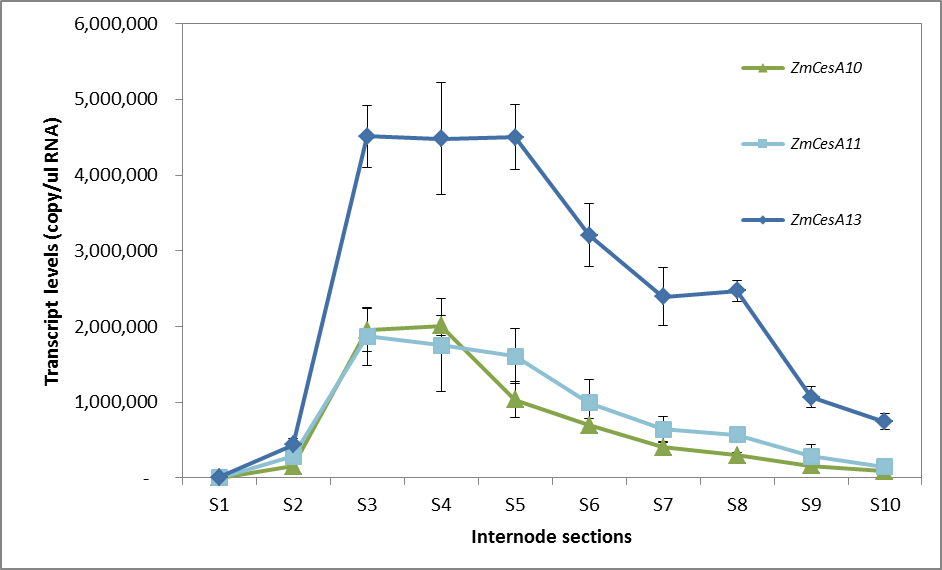


**Supplemental Figure S1** QPCR analysis of secondary wall *CesA* genes

QPCR was conducted with primers list in Table S2 with the same RNA for the microarray experiment. Similar transcript profiles were obtained for the primary wall *CesA* genes from both QPCR and microarray experiments (Figure 4)

**Supplemental Figure S2** The mRNA levels of *Csl* genes


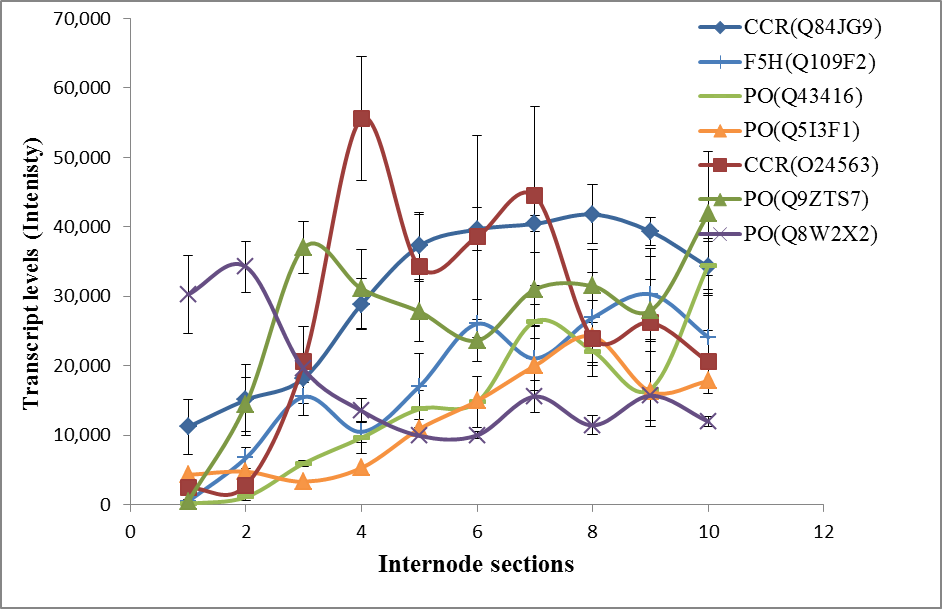


**Supplemental Figure S3** Transcript levels of genes involved in lignin synthesis.
